# Coronary Angiography Characteristics of Symptomatic Patients with Prior Coronary Artery Bypass Graft: A Descriptive Study

**DOI:** 10.1155/2019/1832128

**Published:** 2019-11-11

**Authors:** Xiaolong Ma, Pengfei Chen, Yicheng Zhao, Caiwu Zeng, Meng Xin, Qing Ye, Jiangang Wang

**Affiliations:** ^1^Department of Cardiac Surgery, Beijing Anzhen Hospital, Capital Medical University, Beijing, China; ^2^Center for Cardiac Intensive Care, Beijing Anzhen Hospital, Capital Medical University, Beijing, China; ^3^Department of Cardiac Surgery, Beijing Huaxin Hospital, First Hospital of Tsinghua University, Beijing, China

## Abstract

**Objectives:**

The target of this study was to explore the coronary angiography characteristics for symptomatic patients with prior coronary artery bypass graft (CABG).

**Methods:**

Between 2009 and 2017, 993 patients who had undergone CABG but subsequently suffered recurrent symptoms in Beijing Anzhen Hospital were selected for this study and divided into either medical therapy (MT) group (*n* = 351) or percutaneous coronary intervention (PCI) group (*n* = 642) based on the treatment. Clinical data were analyzed between two groups.

**Results:**

Patients in the MT group were older and more likely to have chronic lung disease (6.6% vs 3.4%, *P*=0.026) while patients in the PCI group were more likely to have prior MI (8.8% vs 17.0%, *P* < 0.001). In the MT group, 54.4% of patients had newly developed lesions both in the graft and native coronary artery while 58.1% in the PCI group (*P*=0.003), and in the MT group, 80.6% had type C coronary artery disease while 60.1% in the PCI group (*P* < 0.001). Patients in the MT group presented higher proportion of diffuse lesions (49.3% vs 15.0%, *P* < 0.001) in native coronary arteries.

**Conclusion:**

Patients receiving MT (35.3%) likely had occluded grafts and type C coronary artery disease featuring as diffuse lesions.

## 1. Introduction

For patients with prior coronary artery bypass graft (CABG), the likelihood of experiencing recurrent symptoms has increased over years due to longer life expectancy [1] and the possibility of disease progression of native coronary artery (NCA) or surgical graft failure [2].

Frequent recurrent symptoms in patients with prior CABG usually indicate the occurrence of myocardial ischemia. Treatments for symptomatic patients with prior CABG include revascularization by percutaneous coronary intervention (PCI) or redo CABG and medical therapy (MT). However, repeat revascularization procedures are markedly different from *de novo* interventions performed on patients without prior CABG [3]. As a consequence of the specificity of vessel lesions and clinical characteristics in patients with prior CABG [4], previous surgery may increase procedural risk, technical complexity [5], and mortality [6, 7], especially for redo CABG. MT consists of antiplatelet agent, statins agent, *β*-blocker, and angiotensin-converting enzyme inhibitor (ACEI) or angiotensin receptor blocker (ARB), considered as optimal medical therapy (OMT) [8]. In fact, clinically, these patients often receive repeat revascularization by PCI for clinical and anatomic factors [2, 9], and patients who could not undergo PCI revascularization would receive MT. However, the coronary angiography (CAG) basis of therapy selection (MT vs PCI) for these patients has been studied in limited data.

In this study, we retrospectively analyzed the clinical data and features of native coronary artery (NCA) lesions and graft lesions by CAG in symptomatic patients with prior CABG who received MT or PCI, aiming to find out CAG characteristics of these patients.

## 2. Methods

### 2.1. Study Design

This study was a retrospective observational study conducted in Beijing Anzhen Hospital, Capital Medical University, Beijing Institute of Heart Lung and Blood Vessel Diseases, Beijing, China. We consecutively included symptomatic patients with prior CABG, from 2008 to 2018 in the Department of Cardiology, Anzhen Hospital. Patients with complete data were segregated into the MT or PCI group, depending on the treatment they received. All data were reviewed by one cardiac surgeon and one cardiologist. This study received approval from the Ethics Committee of Beijing Anzhen Hospital.

### 2.2. Definitions Used in This Study

A graft with the ratio of stenosis diameter to reference vessel diameter ≥70% was defined as stenosis. A graft with stenosis or occlusion was classified as a diseased graft. The classification of ischemic territory was based on CAG results after rehospitalization and also referred to the CAG results prior to CABG. The newly developed lesions were divided into isolated graft disease, NCA and graft disease, and NCA disease.

### 2.3. Statistical Analysis

All results were analyzed by using the statistical package SPSS 20.0. Categorical variables are presented by raw numbers (%), and numerical values are presented as means ± standard deviation. Comparison of the medication and PCI groups was achieved by using Fisher's exact test for each variable and the Mann–Whitney–Wilcoxon nonparametric test for continuous variables. For all analyses reported, *P* values were 2-sided. Statistical differences were considered significant at *P* < 0.05.

## 3. Results

### 3.1. Patient Population

**Figure 1:**
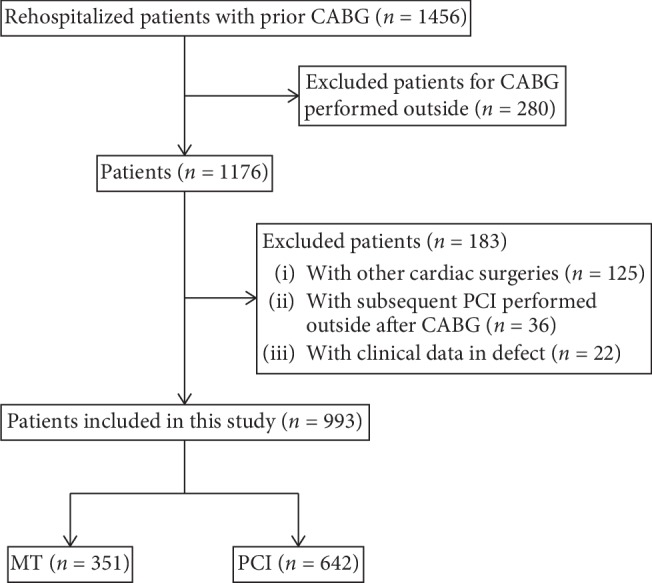
Outline of patients included and classified in this study. CABG = coronary artery bypass graft; CAG = coronary angiography; MT = medical therapy; PCI = percutaneous coronary intervention.

**Figure 2:**
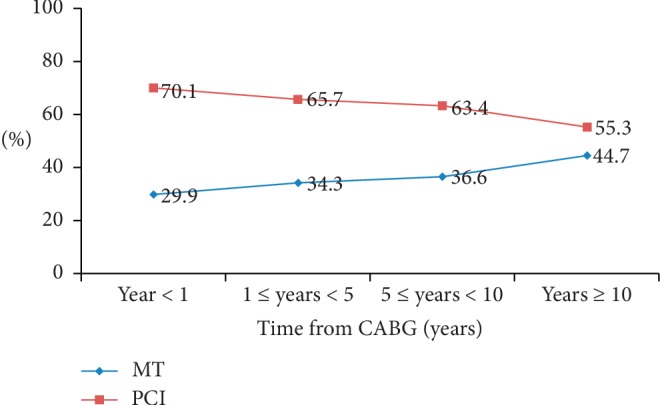
Distribution of patients accepting MT or PCI over different durations after CABG. MT vs PCI *P*=0.014. CABG = coronary artery bypass graft; MT = medical therapy; PCI = percutaneous coronary intervention.

During the study period, 1456 patients with prior CABG were rehospitalized. Based on the exclusion criteria, 993 patients were included in this study (Figure 1). In each group, symptomatic patients with prior CABG were mainly distributed within 1 to 5 years (44.4% vs 46.4%) followed by 5 to 10 years (33.3% vs 31.6%) after CABG surgery (Table 1). The proportion of patients accepting MT increased as a function of time elapsed after CABG while the proportion of patients accepting PCI treatment decreased (Figure 2).

### 3.2. Baseline Characteristics

Baseline characteristics are shown in Table 2. Compared with patients in the PCI group, patients in the MT group were older and more likely to have chronic lung disease while patients in the PCI group were more likely to have prior MI. There were no significant differences in comorbidities like diabetes, hypertension, dyslipidemia, prior PVD, prior CVA, prior HF, or prior PCI between two groups. In each group, most patients had recurrent symptoms featuring as chest pain. In both groups, some patients failed to persist in medical therapy like aspirin, statin, or beta blockers.

### 3.3. CAG Characteristics of Diseased Grafts

The CAG characteristics of diseased grafts are shown in Table 3. There were 480 diseased grafts (stenosis and occlusion) in the MT group, accounting for 46.8% of 1025 grafts, which were lower than those in the PCI group (50.1%). In each group, the proportion of diseased saphenous vein grafts (SVGs) was higher than that of arterial graft, and grafts which were anastomosed at LAD territory and RCA territory were more likely to be diseased. Patients in the MT group were more likely to have chronic total occlusion (CTO) in diseased grafts (82.7% vs 74.8%, *P*=0.003) than those in the PCI group. Due to graft occlusion, there was a high proportion of diseased grafts in each group, in which the location of lesions could not be defined.

### 3.4. CAG Characteristics of Ischemic Territory and Relevant Native Coronary Arteries

Table 4 shows CAG characteristics of the ischemic territories. Patients in the MT group (53.0%) were more likely to suffer one ischemic territory while patients in the PCI group were more likely to suffer two ischemic territories (45.2%); patients in each group were less likely to suffer three ischemic territories (8.8% vs 14.3%), with a significant difference between two groups (*P*=0.001). In one ischemic territory subgroup, in each group, patients were more likely to suffer RCA ischemic territory (36.5% vs 40.8%); in two ischemic territories subgroups, patients were more likely to suffer LCX ischemic territory and RCA ischemic territory (67.2% in the MT group vs 62.8% in the PCI group).

The CAG characteristics of NCA relevant to ischemic territory are also shown in Table 4. In the MT group, 80.6% of patients had type C CAD (severe lesions, based on ACC/AHA Classification of CAD) and no patient had type A CAD, which were obviously different from that in the PCI group (*P* < 0.001). Compared to the PCI group, patients in the MT group were more likely to suffer diffuse lesions (49.3% vs 15.0%, *P* < 0.001). There were no significant differences in CTO lesions, lesions involving coronary artery branches, or openings between two groups.

### 3.5. Supplemental Data of CAG Characteristics and Progression of NCAs

CAG characteristics and progression of native coronary arteries before CABG and at the time of rehospitalization are shown in Supplemental [Supplementary-material supplementary-material-1]. Before CABG, 7.4% of patients had single vessel lesion, 34.5% had two vessel lesions, and 58.1% had three vessel lesions in the MT group; when symptoms recurred after CABG, CAG showed that 2.6% had single vessel lesion, 29.1% had two vessel lesions, and 68.4% had three vessel lesions, which indicated the progression of disease in NCAs. This progression was also observed in the PCI group. There were no significant differences in the number of diseased NCAs between the two groups before CABG or at the time of rehospitalization.

**Figure 3:**
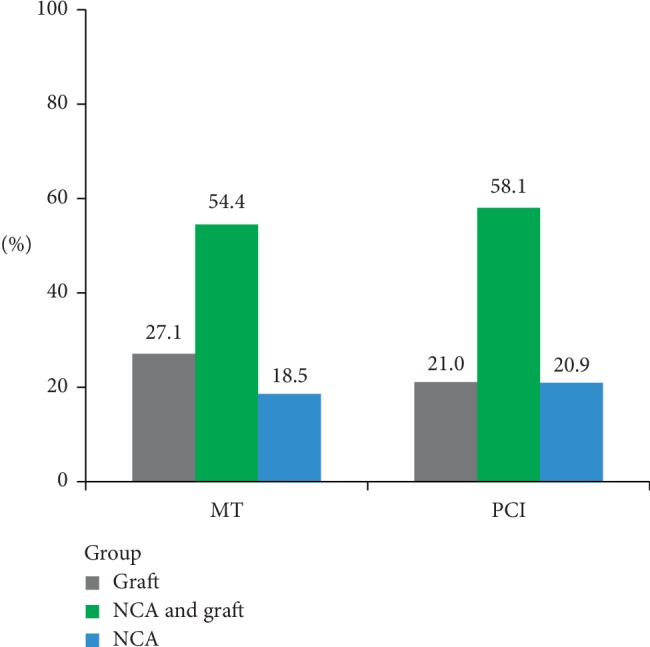
Distribution of patients with newly developed lesions in different vessels. MT vs PCI *P*=0.073. MT = medical therapy; PCI = percutaneous coronary intervention.

Figure 3 shows the distribution of patients with newly developed lesion sites, and there was no significant difference between two groups. In each group, patients were more likely to have newly developed lesions both in NCA and graft, especially in the PCI group (58.1%).

### 3.6. Supplemental Data of PCI Procedural Characteristics

In the PCI group, 86.0% of patients underwent PCI in only NCA and 8.4% underwent PCI in only graft. In patients with ACS, 59 patients (15.5%) underwent PCI in graft, of which 22.0% had PCI in 17 occluded vein grafts (Supplemental [Supplementary-material supplementary-material-1]).

## 4. Discussion

For all we know, this is the largest observational, single-center Chinese study to compare the CAG characteristics of MT versus PCI therapy for symptomatic patients with prior CABG. We find that symptomatic patients with prior CABG are more likely to have newly developed lesions both in the native coronary artery and graft, and patients receiving MT (35.3%) likely had occluded grafts and type C coronary artery disease featuring as diffuse lesions.

The occurrence of myocardial ischemia in patients with prior CABG might be caused by graft failure or disease progression of NCA [10]. Graft lesion rates of SVG and internal mammary artery (IMA) were about 20%–35% and 10%–20% within 5 years following CABG, respectively, and approximately 50%–75% and 20%–40% within 10 years following CABG [2, 11, 12], respectively. Graft failure can be due to conduit defects, anastomotic technical errors, poor native vessel runoff or competitive flow with the native vessel, or atherosclerosis [13]. In this study, 78% of the patients were within 1 to 10 years following CABG, patients included in this study are those with recurrent symptoms after CABG, and most patients have graft stenosis >70% (81.5% in the MT group, 79.1% in the PCI group); therefore, the proportion of patients with graft lesions is high. 44.1% of patients in the MT group and 38.3% of patients in the PCI group have graft age more than 5 years; with the prolongation of time after CABG, the risk of graft disease would also increase. When patients were discharged from hospital after CABG, doctors usually recommended them to take aspirin, beta blockers, and statins throughout their life and control blood pressure and blood sugar. However, in the follow-up, we found that not all the patients in this study did persist in taking these drugs for some reasons like side effects or economic burden, and patients have always been poor in compliance with doctor's advice on medical therapy and lifestyle like exercise, smoking cessation, and low-fat diet. We also observe a high proportion of patients with risk factors associated with atherosclerosis [14], including gender (male), hypertension, diabetes, and hyperlipidemia [15, 16]. Besides, we cannot rule out that the high incidence of graft lesions is associated with the anastomotic technique of surgeons; after all, there is still a gap of medical level and surgical techniques between China and European or American countries.

Redo CABG in patients with prior CABG is more challenging than the first CABG in many aspects [17, 18] including poor basic physical condition, severe pericardial adhesion, and unavailable conduits, which would increase the risk of perioperative complications. Redo CABG is a therapeutic option for some symptomatic patients with prior CBAG, especially in those situations where surgical risk is acceptable; adequate grafts are available, and multiple graft lesions and total occlusions of native coronary arteries and larger amount of ischemic myocardium are present. Compared to redo CABG, PCI had irreplaceable advantages in symptomatic patients with prior CABG, including lower risk of procedural mortality [19, 20], the similar long-term outcome [8], and low medical expense. However, PCI in patients with CTO should be considered against the risk of greater contrast volume, longer fluoroscopy time, and higher MACE rates in comparison with non-CTO patients.

When patients have recurrent symptoms after CABG surgery, they expect to get positive treatment than patients with initial angina, which may be related to their sensitivity to fear and pain of disease. In this study, some patients with stable angina also received PCI, and the choice of therapy depended mainly on doctors but also on the opinions of patients or their family members. Patients receiving MT are older and have more comorbidities; 80.6% had type C lesion featuring as diffuse lesions, which are often companied with extensive atherosclerotic and calcified vessels and longer lesions [21], leading to fewer amenable options for reintervention and suboptimal stent expansion [3]; we reviewed the data of patients with occluded vein grafts in the MT group; cardiologists tried CTO PCI in 22 patients, but all failed at last. Clinically, it was difficult to perform PCI in these patients for high procedural risks and technical-demanding complexity. We did not perform follow-up to these patients who accepted different treatments to further assess outcomes.

## 5. Limitations

Firstly, this study was a retrospective observational study; therefore, it is subject to all the limitations of observational studies. Secondly, the angiography film results were analyzed by one cardiac surgeon and one cardiologist. Thirdly, the classification of graft lesions was in reference to the evaluation criteria of native vessels. Fourthly, the decision to perform PCI for each patient was taken by two operators, based on an evaluation of CAG during CAG. Fifthly, we were just analyzing the clinical characteristics of patients receiving different treatments, especially coronary angiography. We had not yet studied the perioperative complications and follow-up outcomes of these patients treated with different treatments. Sixthly, due to the limitation of medical level and surgical techniques in Beijing Anzhen Hospital in China, we cannot rule out this condition that there might be some unreasonable aspects in surgical techniques and program of the first CABG which are associated with graft failure.

## 6. Conclusions

Symptomatic patients with prior CABG are more likely to have newly developed lesions both in the native coronary artery and graft. Most patients (64.7%) could receive PCI. Patients receiving MT (35.3%) likely had occluded grafts and type C coronary artery disease featuring as diffuse lesions.

## Figures and Tables

**Table 1:** Distribution of patients with recurrent angina accepting different treatments over duration after CABG (*n* = 993).

Variables	MT	PCI	Total
	*n* = 351	*n* = 642	*n* = 993
0-1 yr	40 (11.4%)	94 (14.6%)	134 (13.5%)
1–5 yr	156 (44.4%)	298 (46.4%)	454 (45.7%)
5–10 yr	117 (33.3%)	203 (31.6%)	320 (32.2%)
>10 yr	38 (10.8%)	47 (7.3%)	85 (8.6%)

CABG = coronary artery bypass graft; MT = medical therapy; PCI = percutaneous coronary intervention; yr = year. MT vs PCI *P*=0.147.

**Table 2:** Comparison of baseline patient characteristics of symptomatic patients with prior CABG (*n* = 993).

Variables	MT	PCI	*P* value
	*n* = 351	*n* = 642	
Demographics			
Age (year)	63.65 ± 8.39	62.20 ± 8.81	0.012
≥65 years	166 (47.3%)	262 (40.8%)	0.052
Sex (male)	257 (73.2%)	491 (76.5%)	0.281
Comorbidities			
Diabetes	168 (47.9%)	312 (48.6%)	0.842
Hypertension	269 (76.6%)	482 (75.1%)	0.643
Dyslipidemia	171 (48.7%)	313 (48.8%)	1.000
Chronic renal disease	15 (4.3%)	24 (3.7%)	0.733
Chronic lung disease	23 (6.6%)	22 (3.4%)	0.026
Prior PVD	29 (8.3%)	68 (10.6%)	0.264
Prior CVA	44 (12.5%)	108 (16.8%)	0.080
Prior MI	31 (8.8%)	109 (17.0%)	<0.001
Prior HF	8 (2.3%)	8 (1.2%)	0.291
Prior PCI	15 (4.3%)	33 (5.1%)	0.643
Smoking	208 (59.3%)	412 (64.2%)	0.132
BMI	26.30 ± 3.21	25.91 ± 3.06	0.132
Symptoms			0.415
Chest pain	333 (94.9%)	617 (96.1%)	0.035
SA	142 (42.6%)	236 (38.2%)	
UA	137 (41.1%)	237 (38.4%)	
AMI	54 (16.2%)	144 (23.3%)	
Other	18 (5.1%)	25 (3.9%)	
Mean LVEF%	58.88 ± 10.03	58.69 ± 9.35	0.790
Mean LVEF%^※^	55.24 ± 9.26	56.76 ± 10.94	0.411
Mean LVEF%^§^	59.68 ± 10.32	59.98 ± 7.75	0.780
Duration after CABG	5.11 ± 3.58	4.59 ± 3.42	0.024
Medication after CABG			
Aspirin	289 (82.3%)	504 (78.5%)	0.160
Statin	130 (37.0%)	224 (34.9%)	0.533
Beta blockers	244 (69.5%)	428 (66.7%)	0.394

AF = atrial fibrillation; AMI = acute myocardial infarction; BMI = body mass index; CABG = coronary artery bypass graft; CVA = cerebrovascular accident; HF = heart failure; LVEF = left ventricular ejection fraction; MT = medical therapy; PCI = percutaneous coronary intervention; PVD = peripheral vascular disease; VE = ventricular extrasystole; SA = stable angina; UA = unstable angina; UCG = echocardiography; ^※^results obtained from patients with AMI; ^§^results obtained from patients with SA.

**Table 3:** CAG characteristics of diseased grafts in symptomatic patients with prior CABG (1408 diseased grafts in total 2877 grafts).

Variables	MT	PCI	*P* value
	*n* = 480	*n* = 928	
Diseased grafts	46.8% (480/1025)	50.1% (928/1852)	0.067
Type of diseased graft			0.137
Arterial graft	76 (15.8%)	175 (18.9%)	
SVG	404 (84.2%)	753 (81.1%)	
Anastomosis territory			0.928
LAD territory	167 (34.8%)	321 (34.6%)	
LCX territory	129 (26.9%)	258 (27.8%)	
RCA territory	184 (38.3%)	349 (37.6%)	
Anastomosis type			0.525
Individual	168 (35.0%)	342 (36.9%)	
Sequential	312 (65.0%)	586 (63.1%)	
Lesion characteristics			0.003
CTO	397 (82.7%)	694 (74.8%)	
Stenosis	83 (17.3%)	234 (25.2%)	<0.001
70%–80%	48 (57.8%)	66 (28.2%)	
80%–90%	18 (21.7%)	46 (19.7%)	
90%–99%	17 (20.5%)	121 (51.7%)	
Lesion location			0.002
Aortic anastomosis	61 (12.7%)	131 (14.1%)	
Body	49 (10.2%)	150 (16.2%)	
Distal anastomosis	85 (17.7%)	158 (17.0%)	
Unknown	285 (59.4%)	489 (52.7%)	

CABG = coronary artery bypass graft; CAG = coronary angiography; CTO = chronic total occlusion; LAD = left anterior descending artery; LCX = left anterior descending artery; MT = medical therapy; PCI = percutaneous coronary intervention; RCA = right coronary artery; SVG = saphenous vein graft.

**Table 4:** CAG characteristics of ischemic territory and relevant native coronary arteries in symptomatic patients with prior CABG (*n* = 993).

Variables	MT	PCI	*P* value
	*n* = 351	*n* = 642	
Ischemic territory			0.001
One territory	186 (53.0%)	260 (40.5%)	0.658
LAD	52 (28.0%)	73 (28.1%)	
LCX	66 (35.5%)	81 (31.2%)	
RCA	68 (36.5%)	106 (40.8%)	
Two territories	134 (38.2%)	290 (45.2%)	0.378
LAD + LCX	22 (16.4%)	64 (22.1%)	
LAD + RCA	22 (16.4%)	44 (15.2%)	
LCX + RCA	90 (67.2%)	182 (62.8%)	
Three territories	31 (8.8%)	92 (14.3%)	NS
LAD + LCX + RCA	31 (100.0%)	92 (100.0%)	
Classification of CAD^ψ^			<0.001
Type A	0 (0.0%)	244 (38.0%)	
Type B	68 (19.4%)	9 (1.4%)	
Type C	283 (80.6%)	389 (60.6%)	
Lesion characteristics			
CTO	192 (54.7%)	321 (50.0%)	0.163
Diffuse lesions	173 (49.3%)	96 (15.0%)	<0.001
Branches involved	97 (27.6%)	201 (31.3%)	0.247
Opening involved	97 (27.6%)	195 (30.4%)	0.383

CABG = coronary artery bypass graft; CAD = coronary artery disease; CAG = coronary angiography; CTO = chronic total occlusion; LAD = left anterior descending artery; LCX = left anterior descending artery; MT = medical therapy; NS = not significant; RCA = right coronary artery; ψ = ACC/AHA Classification of CAD.

## Data Availability

The clinical data used to support the findings of this study are included within the supplementary information file.

## Supplementary Materials

Supplementary Materials Supplemental Table 1: CAG characteristics and progression of native coronary arteries before CABG and at the time of rehospitalization. Supplemental Table 2: PCI procedural characteristics of symptomatic patients with prior CABG (*n* = 642).
