# Supplementary material for: Coronary Angiography Characteristics of Symptomatic Patients with Prior Coronary Artery Bypass Graft: A Descriptive Study
Source: Biomed Res Int. 2019 Nov 11;2019:1832128. doi: 10.1155/2019/1832128 (PMC6877980; doi:10.1155/2019/1832128)
Supplement: Supplementary Materials — Supplemental Table 1: CAG characteristics and progression of native coronary arteries before CABG and at the time of rehospitalization. Supplemental Table 2: PCI procedural characteristics of symptomatic patients with prior CABG (n = 642). [file 1832128.f1.pdf]

**Supplemental Table 1** CAG characteristics and progression of native coronary arteries before CABG and at the time of re-hospitalization.

| Variables | MT | PCI | P value |
|---|---|---|---|
| | n=351 | n=642 | |
| Before CABG | | | |
| Diseased vessels | | | 0.672 |
| One vessel | 26(7.4%) | 52(8.1%) | 0.811 |
| LAD | 20(76.9%) | 43(82.7%) | |
| LCX | 3(11.5%) | 5(9.6%) | |
| RCA | 3(11.5%) | 4(7.7%) | |
| Two vessels | 121(34.5%) | 204(31.8%) | 0.753 |
| LAD+LCX | 43(35.5%) | 70(34.3%) | |
| LAD+RCA | 70(57.9%) | 112(54.9%) | |
| LCX+RCA | 8(6.6%) | 22(10.8%) | |
| Three vessels | 204(58.1%) | 386(60.1%) | NS |
| LAD+LCX+RCA | 204(100.0%) | 386(100.0%) | |
| Re-hospitalization | | | |
| Diseased vessels | | | 0.589 |
| One vessel | 9(2.6%) | 15(2.3%) | 0.824 |
| LAD | 7(77.8%) | 10(66.7%) | |
| LCX | 1(11.1%) | 3(20.0%) | |
| RCA | 1(11.1%) | 2(13.3%) | |
| Two vessels | 102(29.1%) | 168(26.2%) | 0.499 |
| LAD+LCX | 36(35.3%) | 66(39.3%) | |
| LAD+RCA | 50(49.0%) | 70(41.7%) | |

| | | | |
|---|---|---|---|
| LCX+RCA | 16(15.7%) | 32(19.0%) | |
| Three vessels | 240(68.4%) | 459(71.5%) | NS |
| LAD+LCX+RCA | 240(100.0%) | 459(100.0%) | |

CABG=coronary artery bypass graft; CAG= Coronary angiography; LAD=left anterior descending artery; LCX=left anterior descending artery; MT= medical therapy; NS=not state; RCA=right coronary artery.

**Supplemental Table 2** PCI procedural characteristics of symptomatic patients with prior CABG(n=642)

| Variable | Patients |
|---|---|
| Target vessel | |
| Target NCA only | 552(86.0%) |
| Target NCA &graft | 36(5.6%) |
| Target graft only | 54(8.4%) |
| Patients with ACS | 381 |
| Target vessel | |
| Target NCA only | 322(84.5%) |
| Target NCA &graft | 24(6.3%) |
| Target graft only | 35(9.2%) |
| Target graft | 59(15.5%) |
| Patients with occluded vein graft | 13 (22.0%) |
| Number of occluded vein grafts | 17 |

ACS= Acute coronary syndrome; CABG=coronary artery bypass graft; CTO=chronic total occlusion; PCI=percutaneous coronary intervention.
